# Pulmonary Artery Root Replacement for Leiomyosarcoma: A Viable Thoracic Oncovascular Operation

**DOI:** 10.7759/cureus.24734

**Published:** 2022-05-04

**Authors:** Papus Keita, Matthew Janko, Nicholas J Peterman, Sandor Toledo, Marc Pelletier

**Affiliations:** 1 Surgery, Carle Foundation Hospital, Urbana, USA; 2 Cardiac Surgery, University Hospitals Cleveland Medical Center, Cleveland, USA; 3 Medicine, The Carle Illinois College of Medicine, Urbana, USA; 4 Surgery, Lehigh Valley Hospital, Allentown, USA

**Keywords:** symptomatic relief, bovine pericardial patch, pulmonary artery, oncovascular, leiomyosarcoma

## Abstract

Leiomyosarcoma of the pulmonary artery is a rare but potentially fatal disease. Due to its rarity, the treatment algorithm is not well-established. While there may be a role for both chemotherapy and radiotherapy, surgical management is the most definitive method. Unfortunately, when the disease process is advanced, surgery may not be curative. However, it may still be a palliative treatment option. In this case report, we present a patient who suffered from respiratory symptoms that were initially attributed to pulmonary embolism (PE). However, upon the diagnosis of pulmonary artery leiomyosarcoma (PAL), surgery intervention was undertaken and resulted in an improved quality of life for the patient.

## Introduction

Malignant primary pulmonary artery tumors are rare entities and infrequently described in the literature. Among these, leiomyosarcomas comprise 21% of all pulmonary artery tumors, making them the most common primary pulmonary artery sarcomas (PPAS) [1]. Nevertheless, the overall incidence of primary pulmonary leiomyosarcomas accounts for only 0.2-0.5% of all lung primary lung malignancies [2]. Therefore, the diagnosis is seldom made timely due to the low index of suspicion, and its presentation is often mistaken for a more common diagnosis such as pulmonary artery thromboembolism [3]. Furthermore, the initial investigation is often non-diagnostic, which complicates the algorithm of care. However, once the diagnosis is suspected, surgical intervention is mandatory for diagnosis, cure, or palliation. In this report, we present a case of pulmonary artery leiomyosarcoma (PAL) that necessitated complete pulmonary root replacement.

## Case presentation

**Figure 1:**
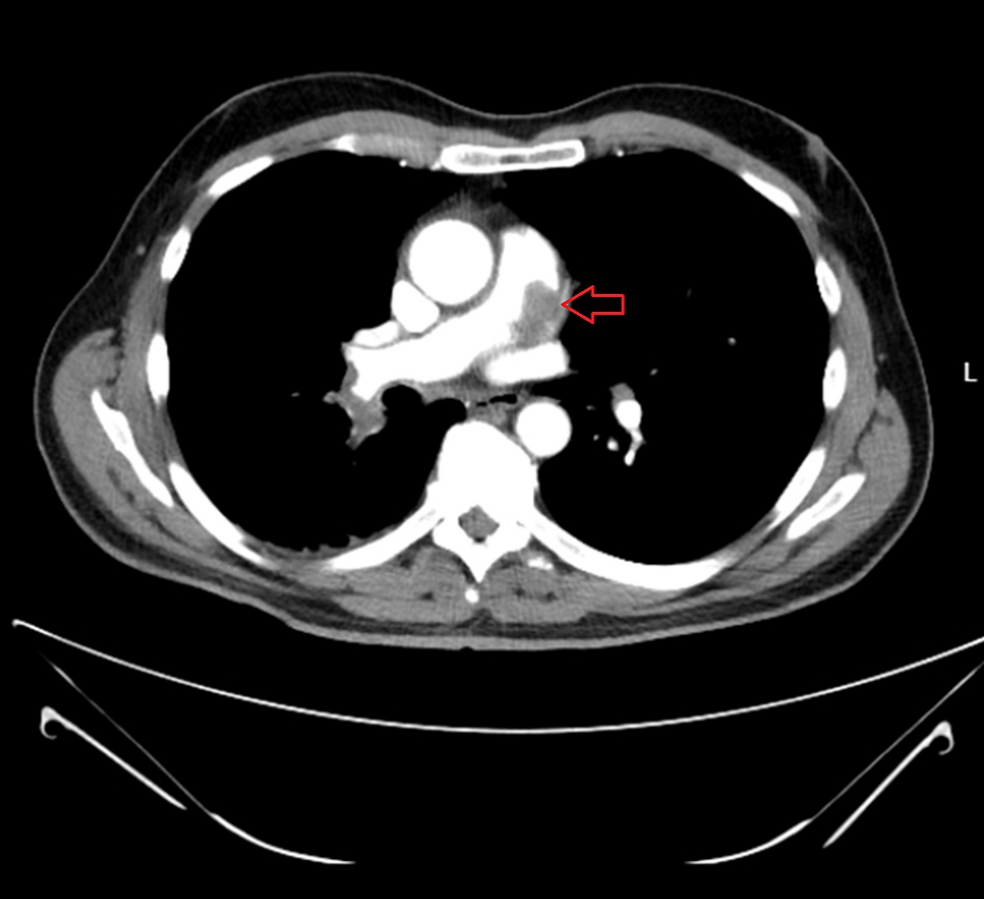
CT pulmonary embolus (PE) with filling defect within the main pulmonary artery (arrow) CT: computed tomography

**Figure 2:**
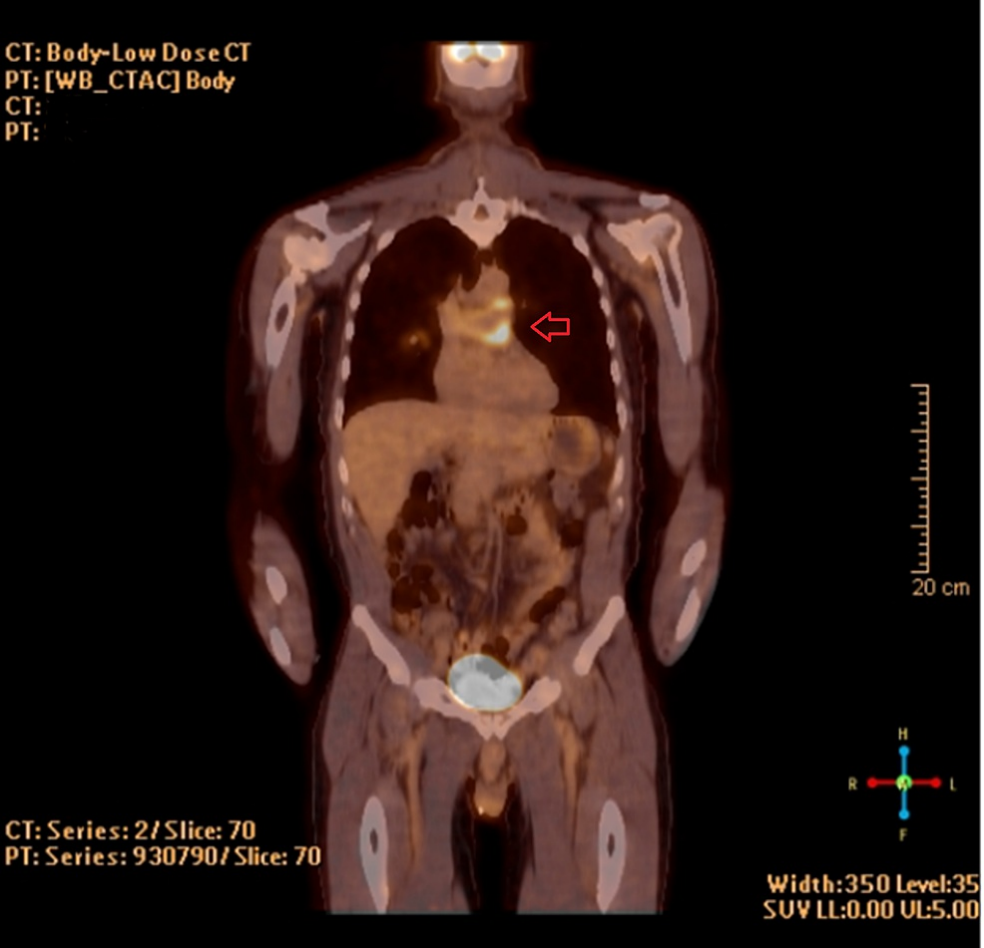
PET/CT demonstrating hypermetabolic lesion within the pulmonary artery and extending bilaterally (arrow) PET: positron emission tomography; CT: computed tomography

A 67-year-old man presented to the cardiac surgery service after undergoing evaluation by the pulmonology team for shortness of breath and cough. He had been symptomatic for several months and was previously diagnosed with a small spontaneous pneumothorax as well as a pulmonary artery embolism, whereafter he was placed on Coumadin. However, the failure to resolve his symptoms in addition to a 10-lb weight loss prompted a CT of the chest, abdomen, and pelvis. An eccentric filling defect at the level of the pulmonary artery was noted (Figure 1). Further investigation with positron emission tomography (PET) demonstrated a hypermetabolic lesion extending from the main pulmonary trunk to the bilateral pulmonary arteries (Figure 2).

**Figure 3:**
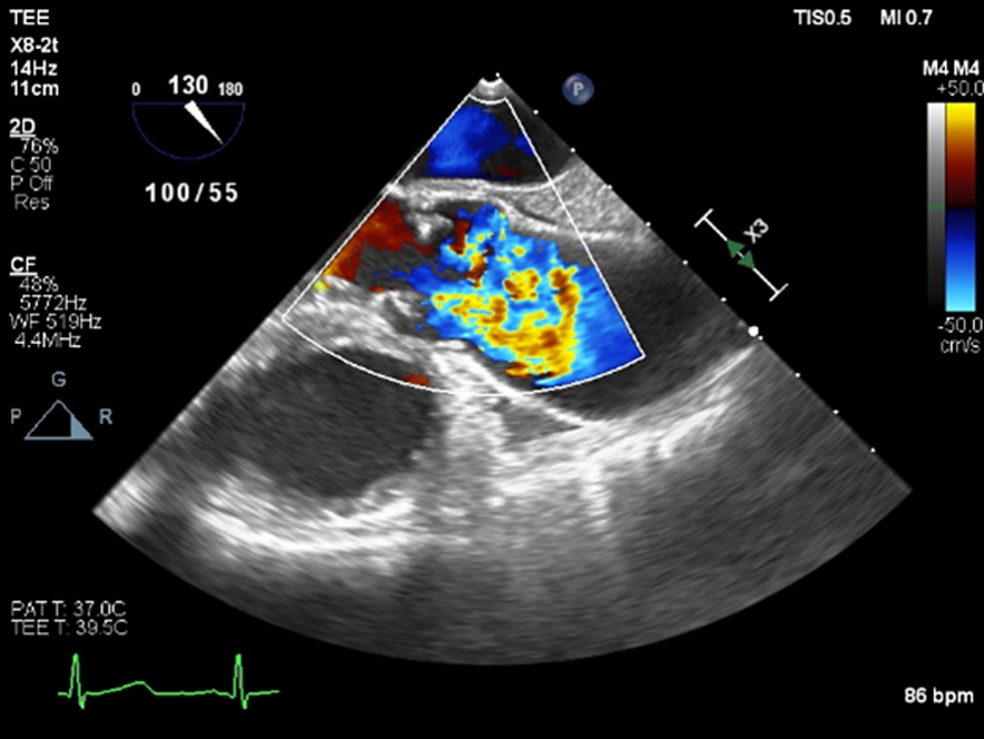
Transesophageal echocardiogram (TEE) demonstrating pulmonic valve insufficiency

**Figure 4:**
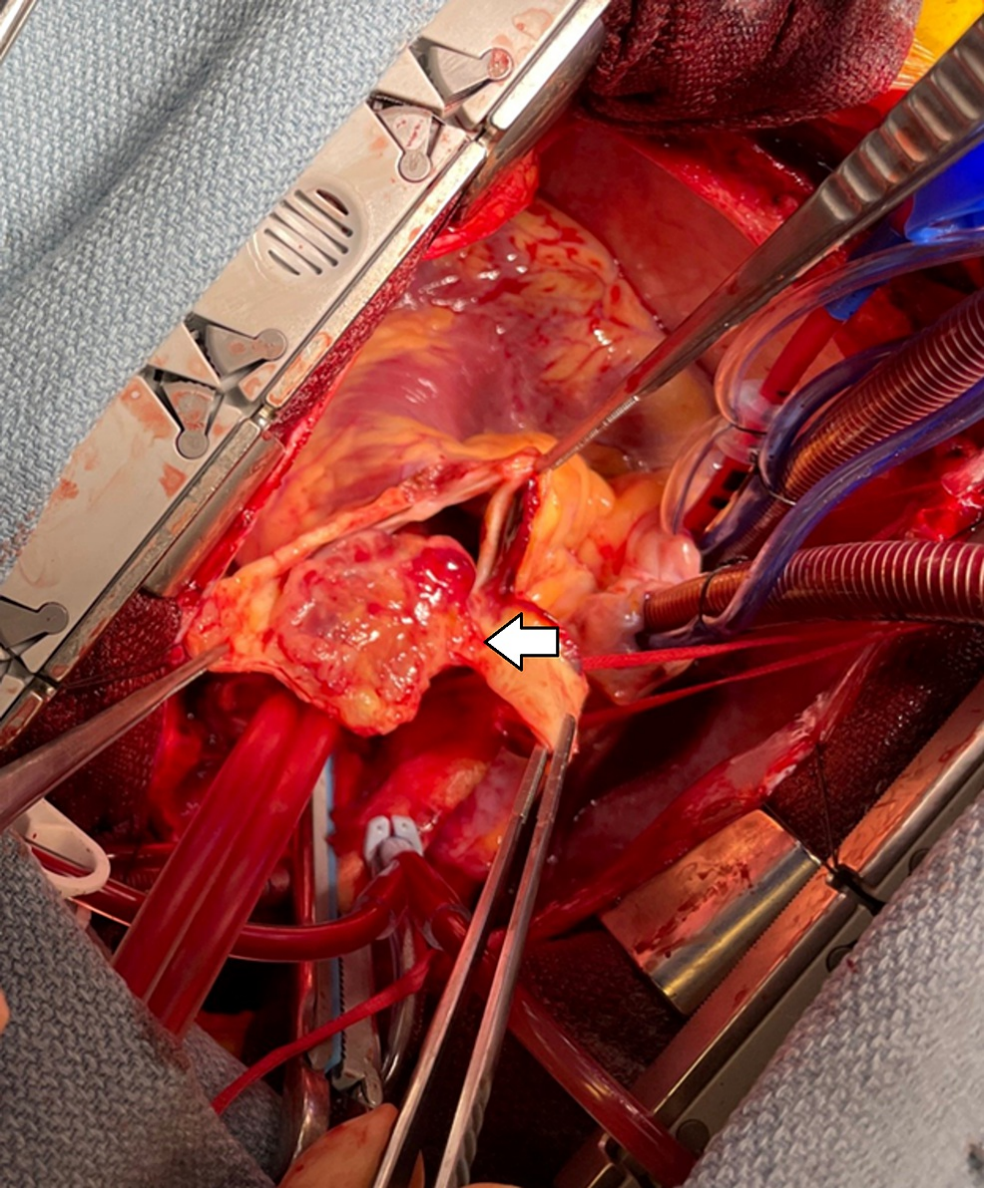
Gross visualization of the pulmonary artery leiomyosarcoma (arrow)

Subsequently, given the high suspicion for malignancy, the patient's condition was discussed at a multi-disciplinary oncology conference where the decision was made to perform surgical resection. Preoperative transesophageal echocardiography (TEE) performed in the operating room showed moderate pulmonic valve insufficiency (Figure 3). After median sternotomy and visual inspection, the tumor was found to be entirely intraluminal and extending into the pulmonary valve. The valve was resected, and the bilateral pulmonary arteries were explored given the preoperative concern for distal tumor extension. Bilateral pulmonary arteriotomies were performed and the tumor was debulked; however, since it was visibly extending into the pulmonary tree on the right side (Figure 4), a complete resection was not possible. The entire pulmonary root was replaced. The decision was then made to augment the caliber of the right pulmonary artery using a bovine pericardial patch. Given the luminal narrowing, it was decided to delay luminal occlusion should tumor regrowth ensue. There were no complications, and the patient tolerated the operation well. He was placed on lifelong oral anticoagulation with warfarin, which has remained within therapeutic range upon follow-up testing.

On postoperative day five, a transthoracic echocardiogram (TTE) was performed before discharge, and the findings were consistent with expected postoperative changes. A pathologic evaluation of intraoperative samples was consistent with grade 3 leiomyosarcoma. One month postoperatively, a chest CT scan demonstrated a stable distal right pulmonary artery tumor burden without recurrence of the resected portions. This was re-demonstrated on cardiac MRI at the six-week interval. The pulmonic valve appeared competent at that time as well. Since that time, the patient’s course has only been complicated by pericardial effusions, which have required both pericardiocentesis and surgical drainage. Notably, cytologic evaluation of the effusion did not demonstrate any malignant cells. Four months postoperatively, he underwent a full course of radiation therapy, which he tolerated well. To date, he has remained alive and improved symptomatically.

## Discussion

The prognosis of PAL is usually poor, with median survival ranging from 1.5 to 12 months after diagnosis [4,5]. Part of the diagnostic dilemma is its rarity, which leads to symptoms being incorrectly attributed to other diagnoses. Therefore, during the initial investigation, the importance of a comprehensive history cannot be understated. For instance, a patient with a low Wells score who has systemic symptoms such as weight loss or chronic cough should be evaluated more extensively. While initial imaging may suggest pulmonary artery thromboembolic disease, the diagnostic algorithm may change depending on the pretest probabilities of an alternative diagnosis.

In a 20-year retrospective study, Bandyopadhyay et al. concluded that in confirmed cases of PPAS, the median time from the onset of symptoms until diagnosis was 100 days [6]. The fact that 47% of patients were initially misdiagnosed with a pulmonary embolism (PE) is especially concerning since the odds of death increase by 46% with each doubling of time from symptom onset to diagnosis [6]. In this case, the elapsed time from the first presentation to the suspected diagnosis was 105 days. Our approach of early operative intervention is best supported by Morreau and Haydock who have reported a six-year survival after aggressive surgical resection [7]. While a cure may not be achievable with many of these tumors, symptomatic relief with subsequent improvement in the quality of life makes early surgical intervention worthy of consideration.

With regard to the operation of choice, Wong et al. have described management options including lobectomy and pneumonectomy but ultimately favored pulmonary endarterectomy as a viable option for symptomatic relief and possible long-term survival [8]. This approach was used in 14 out of 20 patients, which led to symptomatic relief in nearly all patients who underwent the operation [8]. Survival benefits were likely multifactorial given the heterogeneity in perioperative treatment modalities. The remaining six patients had extensive disease burdens that precluded surgical intervention [8]. In our case, the patient’s long-term survival remains to be determined; however, the alleviation of symptoms has been encouraging. Notably, the possibility of pericardial effusion necessitating intervention is worth discussing with patients when considering the overall goals of care. While this may be due to adjuvant radiation rather than surgical intervention, the benefits of radiation in a patient who has undergone an incomplete resection may outweigh such risks.

## Conclusions

PAL is a rare diagnosis, and hence its treatment algorithm is not well elucidated in the literature. While a multi-disciplinary approach is likely best for optimal patient outcomes, surgical interventions may be necessary depending on the overall prognostic outlook. As previously stated, the prognosis of PAL is poor. However, surgical intervention may enhance the quality of life and perhaps improve survival with respect to non-oncologic prognostic measures such as acute cardiopulmonary complications.
